# Financially focused self-concept and problem lottery gambling: the chain mediating role of gambling-related luck beliefs and lottery gambling passion

**DOI:** 10.3389/fpsyt.2026.1978508

**Published:** 2026-10-14

**Authors:** Yue Hu, Lian Liu, Caimei Bai, Zheng Gan, Xiaolong Liu

**Affiliations:** 1 Key Laboratory of Competitive Sport Psychological and Physiological Regulation, School of Education and Psychology, Tianjin University of Sport, Tianjin, China; 2 School of Physical Education, Huzhou Normal University, Huzhou, China; 3 College of Physical Education, School of Sports and Health, Shaanxi Xueqian Normal University, Xian, China; 4 Department of Physical Education & Sport , Central China Normal University, Wuhan, China

**Keywords:** gambling-related luck beliefs, Chinese lottery gambler, financially focused self-concept, gambling passion, problem lottery gambling

## Abstract

**Background:**

China operates the world’s largest lottery market, yet the psychological mechanisms linking individual dispositions to problem lottery gambling remain insufficiently understood, particularly among mainland Chinese samples. Financially focused self-concept—the tendency to base self-worth on financial success—has been linked to gambling problems. However, the pathways linking it to problem lottery gambling have received little empirical attention. This study aimed to investigate the association between financially focused self-concept and problem lottery gambling among Chinese lottery gamblers, and the mediating roles of gambling-related luck beliefs and lottery gambling passion within this association.

**Methods:**

A cross-sectional survey was conducted among Chinese lottery gamblers recruited from land-based retail outlets. A total of 879 participants completed measures of financially focused self-concept, gambling-related luck beliefs, lottery gambling passion, and problem lottery gambling. Structural equation modelling and bias-corrected bootstrapping with 5,000 resamples were used to test the proposed mediation model.

**Results:**

Correlation analyses showed that financially focused self-concept was positively correlated with gambling-related luck beliefs, obsessive passion, harmonious passion, and problem lottery gambling (*p* < 0.001). Financially focused self-concept showed a nonsignificant positive association with problem lottery gambling after accounting for the mediating variables (*β* = 0.03, *p* = 0.40). The specific indirect associations through gambling-related luck beliefs (*β* = 0.034, 95% CI [0.009, 0.061]), obsessive passion (*β* = 0.065, 95% CI [0.036, 0.097]), and harmonious passion (*β* = −0.010, 95% CI [−0.021, −0.001]) were all significant. The serial indirect associations via gambling-related luck beliefs and obsessive passion (*β* = 0.034, 95% CI [0.018, 0.054]) and via gambling-related luck beliefs and harmonious passion (*β* = −0.010, 95% CI [−0.019, −0.004]) were also significant.

**Conclusion:**

Financially focused self-concept is associated with problem lottery gambling through gambling-related luck beliefs, obsessive passion, and harmonious passion. The findings are consistent with the Pathways Model and its recent extension. They also highlight the importance of addressing luck beliefs and internalisation processes in prevention efforts targeting financially focused lottery consumers.

## 1 Introduction

China operates the world’s largest lottery market under a state monopoly. The lottery is the only legal form of gambling in mainland China (1). In 2025, China’s lottery sales totaled RMB 627.97 billion (approximately US$89.7 billion), the highest in the world (2). These sales are supported by a nationwide network of more than 300,000 retail outlets and over 750,000 employees (3). This rapid expansion has brought a growing burden of problem gambling among lottery gamblers. Problem lottery gambling are defined as individuals whose gambling adversely affects themselves and significant others, such as family or friends (4). A 2012 national survey estimated that China had more than 200 million lottery gamblers, and approximately 7 million of them are classified as problem lottery gamblers (5). The proportion of such gamblers rose from about 2% in 2009 (6) to 5% by 2013 (7). Further increases were projected in subsequent years (8). Yet mainland Chinese lottery gamblers have received little empirical attention internationally (9), highlighting the need for targeted research on problem gambling in this population.

Financially focused self-concept refers to the extent to which individuals base their self-definition and self-worth on financial success, specifically the amount of money they possess (10). Individuals high in financially focused self-concept view wealth as the primary measure of personal value and are susceptible to gambling-related harms, including problem lottery gambling. Tabri and Wohl (2021) identified three mechanisms linking financially focused self-concept to problem gambling (11). First, individuals high in financially focused self-concept are strongly motivated to accumulate wealth and are therefore more inclined to gamble for financial gain—a motive that constitutes a well-established risk factor for problem gambling (12). Second, financially focused individuals show steeper delay discounting, favoring immediate rewards over larger delayed ones (13). This immediacy preference may drive loss-chasing behavior, which amplifies financial losses and exacerbates gambling problems (14). Third, individuals high in financially focused self-concept endorse fewer responsible gambling beliefs and engage in fewer responsible gambling behaviors. They take less personal responsibility for their gambling (15, 16) and are less likely to adhere to preset spending limits (17). Empirical work consistently supports the link between financially focused self-concept and problem gambling. In community samples, financially focused self-concept shows a moderate positive correlation with problem gambling, which remains significant after controlling for income, neuroticism, self-esteem, relative deprivation, and materialism (10). Problem gamblers exhibit higher levels of financially focused self-concept than non-problem gamblers (18). Longitudinal evidence over 6–7 months confirms that increases in financially focused self-concept coincide with increases in symptom severity (19).

To date, however, most evidence comes from general gambling or electronic gaming machine samples. The lottery context has received little direct attention. Yet the lottery’s distinctive features may make individuals with a strong financially focused self-concept especially vulnerable. Lottery gambling is often seen as a rare, low-barrier route to rapid financial gain. Its appeal grows under perceived economic disadvantage. For example, experimentally induced perceptions of relative poverty increased lottery gambling behaviors (20). Among Chinese lottery gamblers, relative deprivation strengthened financial gambling motives, especially at lower socioeconomic status (21). The lottery also rewards immediacy. Individuals who prefer smaller, sooner rewards buy more scratch tickets (22). Thus, financially focused self-concept may be especially consequential for problem lottery gambling.

Cognitive distortions play a central role in the development and maintenance of problem gambling (23). Among them, gambling-related belief in luck is especially worth studying in the Chinese cultural context. Superstitious beliefs about luck are widespread among Chinese people (24) and may form a cultural backdrop that heightens and sustains gambling-related harm in this population (25). Such beliefs include the conviction that feng-shui can improve personal luck (26) and the view that numbers such as “6” and “8” carry lucky properties (27). These culturally rooted ideas about luck may encourage and normalize gambling involvement, thereby fostering problem gambling (25, 28). Examining belief in luck among Chinese lottery players therefore carries both theoretical and practical value.

Gambling-related belief in luck refers to the tendency to attribute gambling outcomes to good or bad luck (29). Lottery gamblers holding this belief consider themselves lucky and expect to win sooner or later (30). They overestimate their subjective odds of winning, which keeps them gambling (24, 31). Even after repeated losses, they can construe these outcomes as a sign that luck has not yet arrived rather than as evidence of unfavorable odds. Consequently, they tend to bet more heavily, thereby incurring financial losses that may ultimately lead to problem lottery gambling. This tendency is supported by previous studies. People who believe they are generally lucky gamble more often, wager more money, and report more severe gambling problems (32). Such general beliefs about luck also positively predict participation in national lotteries (33). Evidence specific to gambling contexts points the same way. Longitudinal data show that gambling-related luck beliefs uniquely predict gambling problems both concurrently and at 1- and 6-month follow-ups (28). These beliefs also show stronger links to gambling disorder severity than other gambling-related cognitive dimensions (34), and they prospectively predict gambling relapse (35).

Financially focused self-concept may foster gambling-related luck beliefs. First, people high in financially focused self-concept are often dissatisfied with their finances (36) and feel financially deprived relative to others (11). They are thus more likely to buy lottery tickets to change their situation and regain a sense of control over their finances. Yet the randomness of lottery gambling outcomes and repeated losses frustrate this need for control. This frustration may prompt individuals to turn to external forces and supernatural beliefs to restore a sense of order (37), which in turn fosters stronger gambling-related luck beliefs. Second, financially focused individuals define their self-worth by financial success, and their core motive for gambling is to win money (10). Lottery gambling outcomes, however, are purely random. Under such high uncertainty, the strong pursuit of monetary goals pushes people toward supernatural sources of control. They treat luck as a resource that can be deployed to influence outcomes (30), act in superstitious ways, and prefer lucky objects (38). Through this process, strong gambling-related luck beliefs take shape. Moreover, the monetary motive drives financially focused gamblers to gamble frequently and persistently (12). As their involvement with lottery gambling deepens, gambling-related cognitive distortions, including luck beliefs, develop and become reinforced (23). Tabri et al. (2018) found a significant positive association between financially focused self-concept and gambling-related luck beliefs in a sample of non-problem gamblers (36). Tabri et al. (2021) also found that financially focused self-concept was positively associated with erroneous gambling beliefs, that is, illusion of control and beliefs in luck (39).

Passion is a key concept for understanding why people sustain their engagement in an activity over time. It refers to a strong inclination toward an activity that one likes, values, and invests time and energy in (40). The Dualistic Model of Passion posits that passion emerges when a beloved activity is internalized into one’s identity. The development of passion involves three processes: activity selection, activity valuation, and internalisation. The first two determine the quantity of passion, whereas the mode of internalisation determines its quality (41, 42). Two forms of passion thus arise: harmonious passion stems from an autonomous internalisation, and obsessive passion stems from a controlled internalisation (41, 42). The model has also been applied to gambling (43, 44). Lottery gambling passion refers to a strong inclination toward lottery gambling (45). Obsessive lottery gambling passion arises from a controlled internalisation of lottery gambling into one’s identity and takes the form of an uncontrollable urge to buy lottery tickets (44). Harmonious lottery gambling passion arises from an autonomous internalisation and takes the form of a balanced coexistence of lottery gambling with other life activities (44).

To date, few studies have directly examined the association between financially focused self-concept and lottery gambling passion. Nevertheless, the Dualistic Model of Passion and related empirical findings provide grounds for such a link. First, financially focused gamblers hold a strong monetary motive (10). Such an extrinsic motive tends to drive obsessive passion, whereas its association with harmonious passion is relatively weak (46). Second, people high in financially focused self-concept report lower financial satisfaction, self-esteem, and self-efficacy (11, 36), indicating that their global psychological needs are inadequately met. Among gamblers, Holding et al. (2021) found that lower global need satisfaction was associated with higher obsessive passion (47). In addition, from the perspective of passion quantity, the monetary motive drives financially focused gamblers to gamble frequently and persistently (10). As lottery gambling becomes more central to their lives, this deepening involvement may also foster harmonious passion.

Similar to the case of financially focused self-concept and passion, few studies have directly examined the association between gambling-related luck beliefs and lottery gambling passion. First, gambling-related luck beliefs may increase both harmonious and obsessive passion by deepening gambling involvement. Studies show that gamblers who believe in luck gamble more frequently and spend more money (48–50). According to the Dualistic Model of Passion (41), as gambling involvement deepens, lottery gambling becomes increasingly important and central in their life, which in turn strengthens both forms of passion. Second, gambling-related luck beliefs may foster obsessive passion through controlled internalisation. Gamblers with strong luck beliefs tend to incorporate luck into their self-concept and see themselves as inherently lucky (51, 52). They also treat luck as a personal possession that they can draw upon (53). Meanwhile, luck beliefs imply that persistence will ultimately lead to winning (54). Consequently, these gamblers continue buying tickets after losses to validate their luck. Lottery gambling thus becomes a means of testing luck, generating an internal pressure that drives obsessive passion (40). In addition, gambling-related luck beliefs may also promote harmonious passion. General beliefs about being lucky sustain optimism, confidence, and reduced anxiety in the face of uncontrollable events (55–57). Gambling-related luck beliefs are the manifestation of this trait in the gambling context (53). Such beliefs heighten the joy of winning and ease the frustration brought by losses. This relatively pressure-free engagement facilitates autonomous internalisation, thereby promoting harmonious passion.

In addition, the two forms of lottery gambling passion are expected to relate differently to problem lottery gambling. Harmonious passion arises from intrinsic rewards, such as enjoyment and challenge. Gamblers with this orientation keep lottery gambling in balance with other life domains. The activity yields mostly positive experiences, including pleasure and relaxation (45, 58). In this case, the person retains control over the activity, rather than being controlled by it. Harmonious passion is therefore expected to be negatively associated with problem lottery gambling. Obsessive passion, by contrast, stems from internal pressure—for instance, to win money or escape difficulties (59). Here, the activity comes to control the individual. Such rigid persistence makes it difficult to stop even as losses accumulate (40). Excessive engagement may consequently escalate into problem lottery gambling. Empirical findings support this division. In a large sample of Chinese lottery gamblers, obsessive passion was positively associated with problem lottery gambling, whereas harmonious passion showed a negative association (3). A scoping review of 17 studies concluded that obsessive passion is consistently linked to gambling problems, riskier betting patterns and stronger gambling intentions, whereas harmonious passion is mostly unrelated to such outcomes (60). Likewise, a recent study found that, after shared variance was controlled, harmonious passion showed a weak negative association with gambling severity, whereas obsessive passion showed a strong positive association (61).

In summary, financially focused self-concept has been associated with gambling problems, yet its association with problem lottery gambling and the underlying psychological mechanisms remain poorly understood. The current study addresses this gap in a sample of Chinese lottery gamblers. We aimed to clarify how financially focused self-concept is associated with problem lottery gambling in this population and to test a statistical mediation model in which gambling-related luck beliefs and lottery gambling passion (harmonious and obsessive) were modelled as mediators of this association. Specifically, we aimed to: (a) examine the direct association between financially focused self-concept and problem lottery gambling (Hypothesis 1); (b) test the mediating role of gambling-related luck beliefs in this association (Hypothesis 2); (c) test the mediating roles of harmonious and obsessive lottery gambling passion in this association (Hypothesis 3); and (d) test whether gambling-related luck beliefs and lottery gambling passion sequentially mediate this association (Hypothesis 4).

## 2 Methods

### 2.1 Participants

Given that online lottery gambling is prohibited in mainland China, recruitment was conducted at land-based lottery retail outlets. A total of 72 retail outlets were randomly selected from Zhejiang, Yunnan, and Hainan provinces. Eight trained research assistants were involved in participant recruitment. They approached the participants on site and informed them of the study purpose, the voluntary nature of participation, and their right to withdraw from the survey at any time without penalty. The research assistants then invited them to complete an anonymous paper questionnaire. Participants were eligible if they were at least 18 years of age and had purchased lottery tickets within the past 12 months (62). The questionnaire took approximately 5–10 minutes to complete. Written informed consent was obtained prior to enrolment. Formal ethics approval for this study was granted by Tianjin University of Sport’s Human Research Ethics Committee (2026–085). After completing the study, participants were remunerated with two lottery tickets of nominal value (approximately 4 RMB in total). Participants who preferred not to receive lottery tickets were offered cash of equivalent value instead. The compensation was given to all participants and was not contingent on the content of their responses.

Of the 900 returned questionnaires, 21 (2.33%) were excluded based on two prespecified data-quality criteria. The first was extensive blank items, defined as more than 10% of items left unanswered. The second was stereotyped answering patterns, defined as endorsing the same response option for at least 80% of consecutive items across the questionnaire. These criteria are consistent with established conventions for identifying careless or insufficient-effort responding (63, 64). The final sample comprised 481 male (54.7%) and 398 female participants (45.3%). The age range of the participants was 18 to 71 years (*M*age = 32.55 years, *SD* = 10.89). Participants also reported the lottery game type on which they spent the most: number games (406, 46.2%), scratch cards (328, 37.3%), and sports betting (145, 16.5%). For retained cases, missing values were handled by mean substitution before analysis.

### 2.2 Measures

#### 2.2.1 Financially focused self-concept

Financially Focused Self-Concept was measured with the Short Financially Focused Scale (10). We translated the scale into Chinese following standard forward- and back-translation procedures. Two bilingual researchers independently translated the items into Chinese, then compared and reconciled their versions through discussion to produce a single Chinese version. An independent bilingual translator, blinded to the original, back-translated this version into English. We compared the back-translated version with the original item by item and refined the wording until semantic equivalence was achieved. Finally, gambling psychology experts reviewed the Chinese version, and we piloted it with a small sample of Chinese lottery gamblers to ensure item clarity and comprehensibility. The scale consists of 4 items (e.g., ‘How I feel about myself is largely based on the amount of money I have’) referring to the past 12 months. It was rated on a 5-point scale ranging from 0 (not at all) to 4 (extremely). The result of confirmatory factor analysis showed an overall acceptable fit to the data: *χ*^2^ = 10.32, *df* = 2, *p* = 0.006, *χ*^2^/df = 5.16; *RMSEA* = 0.07; *TLI* = 0.99; *CFI* = 0.99. The significant χ² value reflects the sensitivity of χ² to the large sample size (*n* = 879); all approximate fit indices indicated good fit. Standardized factor loadings ranged from 0.79 to 0.90 (all *ps* < 0.001). The Cronbach’s α coefficient was 0.81 in this study.

#### 2.2.2 Gambling-related luck beliefs

Gambling-Related Luck Beliefs was measured with the luck subscale in the Gambling Attitudes and Beliefs Survey (GABS-23) (29). We translated it into Chinese following the same procedures. The luck beliefs subscale includes four items, such as “Some people have bad luck when buying lottery tickets.” It was rated on a five-point scale ranging from 1 (strongly disagree) to 5 (strongly agree). The result of confirmatory factor analysis showed an overall acceptable fit to the data: *χ*^2^ = 3.34, *df* = 2, *p* = 0.19, *χ*^2^/df = 1.67; *RMSEA* = 0.03; *TLI* = 0.99; *CFI* = 0.99. Standardized factor loadings ranged from 0.67 to 0.86 (all *ps* < 0.001). The Cronbach’s α was 0.78.

#### 2.2.3 Lottery gambling passion

Lottery gambling passion was measured with a ten-item Gambling Passion Scale (43). A translated Chinese version of the Gambling Passion Scale has been validated (3). The scale consisted of two sub-scales: harmonious passion (e.g., “Lottery Gambling is in harmony with the other activities in my life”) and obsessive passion (e.g., “I cannot live without lottery gambling”). Each sub-scale contained 5 items and was rated on a 7-point scale ranging from 1 (strongly disagree) to 7 (strongly agree), with higher scores indicating stronger gambling passion. The Cronbach’s α coefficient for harmonious passion and obsessive passion was 0.91 and 0.97, respectively.

#### 2.2.4 Problem lottery gambling

Problem lottery gambling was measured by the Problem Gambling Severity Index developed by Ferris and Wynne (4). A translated Chinese version of the Problem Gambling Severity Index has been validated (3). It included 9 items, such as “bet more than you could afford to lose”. Each item is rated on a 4-point Likert scale from 0 (never) to 3 (almost always), with higher scores indicating a greater risk for problem gambling. The Cronbach’s α coefficient was 0.95 in this study.

### 2.3 Data analysis

All statistical analyses were performed using SPSS 22.0 and AMOS 31.0. Descriptive statistics are reported as means and standard deviations. To assess potential common method bias arising from the use of self-report measures, we conducted Harman’s single-factor test. Pearson correlation analyses were performed to examine bivariate associations among the main variables. We employed confirmatory factor analysis to evaluate the validity of the translated scales. The hypothesized chain mediation model was estimated as a latent-variable structural equation model in AMOS 31.0. Financially focused self-concept, gambling-related luck beliefs, harmonious passion, obsessive passion, and problem lottery gambling were each modelled as a latent variable indicated by their individual items. Each latent factor was identified by fixing one factor loading to 1. The disturbances of harmonious passion and obsessive passion were allowed to covary, given the theoretical relatedness of the two constructs. Sex, age, and gambling type were included as observed covariates predicting all endogenous constructs, so that both the direct and indirect paths were adjusted for these demographic and gambling-context characteristics. Gambling type was dummy-coded into two indicator variables with number games as the reference category. Parameters were estimated using maximum likelihood, and indirect effects were tested with bootstrapping based on 5,000 resamples. Model fit was assessed via *χ*^2^, *CFI*, *TLI*, *RMSEA*, and *SRMR.* CFI, TLI ≥ 0.90, and RMSEA, *SRMR* ≤ 0.08 indicate acceptable fit. Indirect effects were examined with bias-corrected bootstrapping based on 5,000 resamples. An effect was regarded as significant when its 95% confidence interval did not include zero. Standardized coefficients are reported for all paths. All tests were two-tailed, and the significance level was set at *p* < 0.05.

## 3 Results

### 3.1 Common method bias test

We implemented procedural strategies to reduce the risk of common method variance. Participation was anonymous, and respondents were informed that no responses were right or wrong, which was intended to encourage honest answering and minimize social desirability bias (65). We then conducted Harman’s single-factor test. The unrotated factor solution yielded 5 factors with eigenvalues above 1.0, with the first factor explaining 25.52% of the total variance—well below the 40% benchmark. This result suggests that common method bias was not a significant threat to our data.

### 3.2 Descriptive statistics and correlation analysis

Descriptive statistics and correlations are presented in **Table 1**. Financially focused self-concept exhibited a positive correlation with gambling-related luck beliefs, obsessive passion, harmonious passion, and problem lottery gambling. Gambling-related luck beliefs were positively related to obsessive passion, harmonious passion, and problem lottery gambling. Obsessive passion and harmonious passion were also correlated positively with problem lottery gambling.

**Table 1:** Results of descriptive statistics and correlation analysis.

Variable	*M*	*SD*	1	2	3	4	5
1.Financially Focused Self-Concept	2.18	0.89	1.00				
2.Gambling-Related Luck Beliefs	3.16	0.78	0.48^***^	1.00			
3.Obsessive passion	2.56	1.52	0.27^***^	0.25^***^	1.00		
4.Harmonious passion	3.54	1.53	0.21^***^	0.23^***^	0.54^***^	1.00	
5.Problem lottery gambling	0.51	0.68	0.22^***^	0.22^***^	0.51^***^	0.20^***^	1.00

*** *p* < 0.001. *M*, Mean; *SD*, Standard deviation.

### 3.3 Mediation analysis of gambling-related luck beliefs and lottery gambling passion

A structural equation model was performed to analyze the mediating roles of gambling-related luck beliefs and lottery gambling passion in the association between financially focused self-concept and problem lottery gambling. Results showed that the model fit indices were all within the acceptable range: *χ*^2^ = 1453.72, *df* = 412, *χ*^2^/df = 3.53, *p* < 0.001; *RMSEA* = 0.05; *SRMR* = 0.05; *TLI* = 0.95; *CFI* = 0.95. Standardized factor loadings of all constructs were statistically significant (ps <.001), ranging from.69 to.95 (see **Supplementary Table S1**). **Figure 1** presents the standardized path coefficients for the chain mediation model. Financially focused self-concept showed a nonsignificant positive association with problem lottery gambling (*β* = 0.03, *p* = 0.40). We estimated indirect effects via bootstrapping with 5,000 resamples (**Table 2**). An indirect effect was considered significant when its bias-corrected 95% confidence interval excluded zero. The specific indirect effects through gambling-related luck beliefs (*β* = 0.034, 95% *CI* [0.009, 0.061]), obsessive passion (*β* = 0.065, 95% *CI* [0.036, 0.097]), and harmonious passion (*β* = −0.010, 95% *CI* [-0.021, -0.001]) were all significant, supporting Hypotheses H2 and H3. The two serial indirect paths—via gambling-related luck beliefs and obsessive passion (*β* = 0.034, 95% *CI* [0.018, 0.054]), and via gambling-related luck beliefs and harmonious passion (*β* = −0.010, 95% *CI* [-0.019, -0.004])—were also significant, providing support for Hypothesis H4.

**Figure 1:**
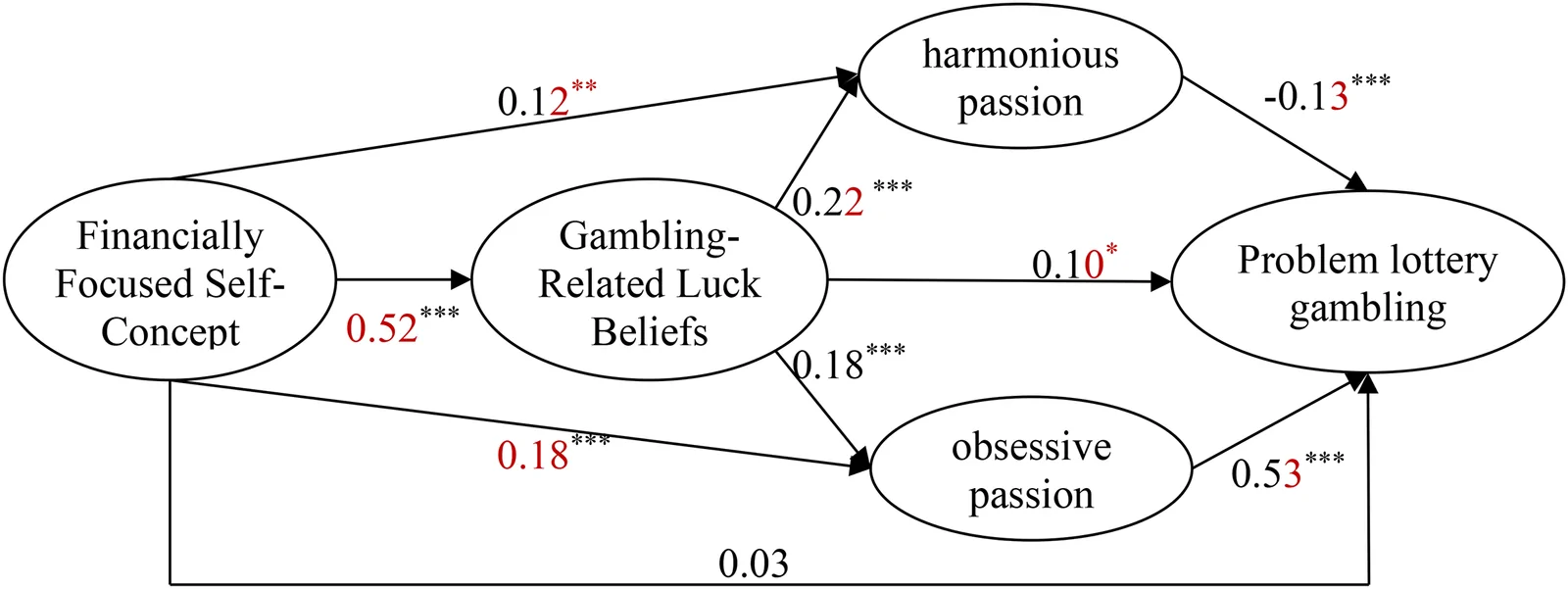
Chain mediating effect model of gambling-related luck beliefs and lottery gambling passion between financially focused self-concept and problem lottery gambling. Values are standardized regression coefficients (*β*); ^*^p < 0.05, ^**^p < 0.01, ^***^p < 0.001.

**Table 2:** Results of the mediating effect.

Path	*β*	SE	95% CI
Direct effect			
financially focused self-concept → problem lottery gambling	0.031	0.034	-0.036, 0.097
Indirect effect			
financially focused self-concept →gambling-related luck beliefs → problem lottery gambling	0.034	0.013	0.009, 0.061
financially focused self-concept →obsessive passion → problem lottery gambling	0.065	0.015	0.036, 0.097
financially focused self-concept →harmonious passion → problem lottery gambling	-0.010	0.005	-0.021, -0.001
financially focused self-concept →gambling-related luck beliefs →obsessive passion →problem lottery gambling	0.034	0.009	0.018, 0.054
financially focused self-concept →gambling-related luck beliefs →harmonious passion →problem lottery gambling	-0.010	0.004	-0.019, -0.004
Total indirect effect	0.114	0.020	0.078, 0.154
Total effect	0.135	0.022	0.092, 0.177

Values are standardized regression coefffcients (*β*); *CI*, conﬁdence interval; 95% CI was based on bootstrap with 5,000 resamples; *SE*, standard error.

## 4 Discussion

The present study aimed to examine the association between financially focused self-concept and problem lottery gambling among Chinese lottery gamblers and to test three mediating mechanisms: gambling-related luck beliefs, lottery gambling passion, and their sequential combination. Four key findings emerged. First, consistent with Hypothesis 1, gamblers who grounded their self-worth in financial success tended to report more severe problem lottery gambling. Second, as predicted by Hypothesis 2, gambling-related luck beliefs mediated this association. Third, in line with Hypothesis 3, both forms of passion also mediated this association but in opposite directions: the indirect association through obsessive passion was positive, whereas that through harmonious passion was negative. Fourth, the two serial indirect associations—via luck beliefs and then each form of passion—were also significant and again operated in opposite directions, supporting Hypothesis 4. Notably, the direct association between financially focused self-concept and problem lottery gambling was no longer significant when these mediators were included in the model. We discuss these findings below.

### 4.1 Correlation between financially focused self-concept and problem lottery gambling

In an era of global economic uncertainty and widening income inequality, financial success has become a central metric of self-worth for many. Individuals who ground their self-worth in financial success report greater financial social comparison, financial hassles, stress, and anxiety (66). A lottery ticket costs little but offers a chance of instant wealth—a combination that leads many to perceive it as a rare and affordable means of substantially improving their standard of living (67). This makes lottery gambling appealing to those who ground their self-worth in financial success, especially individuals facing economic disadvantage (21) and those with a preference for immediate rewards (22).

This study found that financially focused self-concept was positively correlated with problem lottery gambling, which supports Hypothesis 1. This finding aligns with prior work. Financially focused self-concept has been linked to greater gambling involvement and problem gambling severity across several gambling forms (10, 18, 36). Lottery gamblers with a strong financially focused self-concept are often dissatisfied with their financial situation. They exhibit strong financial motives for gambling (12), show poor self-regulation in their betting behavior (17), and invest excessive money, which tends to amplify losses and heighten the risk of problem gambling.

Notably, the direct association between financially focused self-concept and problem lottery gambling was no longer significant when gambling-related luck beliefs and gambling passion were entered into the model. This pattern suggests that financially focused self-concept is better understood as a distal rather than a proximal correlate of problem lottery gambling. As a domain-general self-concept, financially focused self-concept may need to be translated into lottery-specific psychological states before it shapes behavior. This account aligns with the view that broad personal vulnerabilities are linked to outcomes through proximal motivational and cognitive processes (12). Additionally, the model reveals opposing indirect pathways: financially focused self-concept was positively associated with harmonious passion, which was in turn negatively associated with problem lottery gambling. This opposing pattern of indirect associations may partly account for the weak residual direct association.

### 4.2 Mediating role of gambling-related luck beliefs

The present study found a significant indirect association between financially focused self-concept and problem lottery gambling through gambling-related luck beliefs, supporting Hypothesis 2. Financially focused gamblers are often dissatisfied with their finances, and repeated losses make them prone to a sense of control deprivation (11, 36). They then tend to turn to external forces and supernatural beliefs to restore a sense of order (37), a tendency associated with stronger gambling-related luck beliefs. Gamblers who believe that luck can influence winning outcomes tend to rationalize their wins and losses *post hoc*. A win is attributed to personal good luck, and gamblers feel they should continue to gamble to take advantage of their good luck. A loss is attributed to temporary bad luck, which keeps hope for the next draw alive. Such reasoning may sustain continued gambling irrespective of whether outcomes are wins or losses (30, 48). Over time, this persistent engagement may coincide with weakened behavioral control, a pattern characteristic of problem lottery gambling.

These findings carry a broader message. Attitudes and values formed outside the gambling domain may be associated with irrational beliefs within it, and these beliefs are in turn associated with problem gambling. This pattern is consistent with the Pathways Model, which treats erroneous beliefs about gambling as a core process in the development of problem gambling (23). It also aligns with the recent extension of that model, in which a financially focused self-concept and erroneous gambling beliefs are jointly proposed as core psychopathologies of problem gambling (68). Our results provide empirical support for this proposal in a large sample of Chinese lottery gamblers. They also converge with earlier evidence that financially focused self-concept correlates positively with gambling-related beliefs in luck (11, 36).

### 4.3 Mediating role of lottery gambling passion

At the bivariate level, both obsessive passion and harmonious passion correlated positively with problem lottery gambling. However, their unique associations diverged in the model: obsessive passion was positively associated with problem lottery gambling, whereas harmonious passion showed a negative unique association. This reversal is unsurprising. This sign reversal should be interpreted with caution. The two forms of passion were substantially correlated, and both reflect a strong inclination to lottery gambling. The bivariate correlation of harmonious passion with problem lottery gambling may therefore partly reflect this shared variance. After accounting for obsessive passion, harmonious passion showed a negative conditional association. This pattern is consistent with statistical suppression, in which an association reverses sign once the variance shared with a correlated variable is accounted for (69). Theoretically, however, a genuinely protective role of harmonious passion is also plausible. Harmonious passion is grounded in autonomous internalisation, which is characterized by flexible engagement that remains in balance with other life domains. Such engagement may be less likely to be associated with conflict and harm. This result aligns with previous studies, in which harmonious passion generally shows null or modest positive bivariate correlations with problem gambling, and its protective role becomes apparent only when obsessive passion is statistically controlled (3, 43, 45, 46, 61). The present cross-sectional data cannot adjudicate between the two accounts, and longitudinal or experimental evidence is needed.

Our results further revealed that harmonious and obsessive passion each carried an independent indirect association between financially focused self-concept and problem lottery gambling. Specifically, the indirect association through obsessive passion was positive and significant, whereas that through harmonious passion was negative and significant, with the indirect association through obsessive passion clearly the stronger of the two. By tying self-worth to lottery outcomes and leaving basic psychological needs unsatisfied (47), financially focused self-concept is associated with a tendency toward controlled rather than autonomous internalisation. This tendency is linked to stronger obsessive passion (42), which is in turn associated with deeper gambling involvement and, ultimately, problem lottery gambling. Money-driven involvement is also associated with greater overall gambling volume, which may partly account for the positive association with harmonious passion. The autonomous and flexible nature of harmonious internalisation, in contrast, may account for its negative unique association with problem lottery gambling.

The serial indirect associations through gambling-related luck beliefs and passion exhibited the same opposing pattern. The indirect association through luck beliefs and obsessive passion was positive and significant, whereas that through luck beliefs and harmonious passion was smaller and negative. The divergence between the two chains appeared to lie downstream rather than upstream. Luck beliefs were positively associated with harmonious passion and obsessive passion. The marked difference lay in how the two passion forms related to problem lottery gambling: positively for obsessive passion and negatively for harmonious passion. In other words, luck beliefs showed associations with both passion forms, with no strong directional preference. This pattern is consistent with the Dualistic Model of Passion, in which the mere valuation of and engagement in an activity sustains passion generally, whereas the type of internalisation determines whether passion takes a harmonious or obsessive form (40, 44). Luck beliefs have been linked to greater gambling involvement (28, 33), which may be in turn associated with the development of both passion forms. In non-gambling domains, feeling lucky has been shown to foster optimism, confidence, and positive outcome expectations (70), as well as a sense of psychological security (71). Luck beliefs may be associated with greater intrinsic enjoyment of lottery gambling, which may in turn be associated with harmonious passion. This may help explain why the association between luck beliefs and harmonious passion was slightly more pronounced than that with obsessive passion.

### 4.4 Practical implications

These findings carry implications for lottery regulation and public education in the Chinese context. Although lottery publicity in mainland China has already been steered away from jackpot-centered messaging, and marketing that links lottery gambling to overnight riches, wealth management, or financial advancement has been curtailed, a considerable proportion of lottery gamblers still purchase tickets to make money or win a large prize (12). This motive appears especially relevant for individuals who ground their self-worth in financial success, the group that showed the strongest links to problem lottery gambling in this study. Regulatory messaging could therefore go further. Rather than merely reducing wealth-related appeals, publicity could state directly that lottery gambling cannot bring about a change in personal wealth. Such messaging could also address the *post hoc* rationalization of wins and losses documented here, making explicit that each draw is independent and that luck can be neither accumulated nor exhausted. For the financially focused group identified here, interventions could additionally help gamblers decouple self-worth from financial success.

These findings can inform lottery regulation and public education in China. Lottery gambling regulators in mainland China have moved away from jackpot-centered messages. Marketing that links lottery gambling to getting rich quickly or improving personal finances has also been limited. Yet many lottery gamblers still buy tickets to make money or win a large prize (12). This motive may be especially relevant for people who base their self-worth on financial success. In this study, this group showed a positive association with problem lottery gambling. Regulators could go further. Instead of only reducing wealth-related appeals, publicity could state that lottery gambling will not change personal wealth. Such messages could also address how gamblers explain wins and losses after the fact. They could state that each draw is independent and that luck cannot be accumulated or exhausted. For the financially focused group in this study, interventions could also help gamblers separate self-worth from financial success.

The findings also have implications for prevention and intervention. The association between financially focused self-concept and problem lottery gambling was mediated by gambling-related luck beliefs. Cognitive interventions that target distorted gambling beliefs are a core component of effective treatments for problem gambling (31, 72). Such interventions could be adapted for lottery gamblers, who are rarely reached by clinic-based services. These interventions could also be tailored to individuals. Lottery gamblers differ in the specific beliefs they hold. A brief assessment of luck-related beliefs could allow psychoeducation and cognitive correction to be tailored to each gambler’s belief profile, rather than delivered in a generic form.

Finally, the two forms of passion showed opposite associations. This suggests that prevention should consider how individuals engage with lottery gambling, not only how much they gamble. Public education could stress the recreational nature of lottery gambling and highlight signs of obsessive passion, such as gambling to maintain self-worth or to escape discomfort. Helping gamblers recognize these signs could enable earlier prevention, before controlled engagement becomes entrenched. Rigid engagement tied to self-worth marks elevated risk, whereas flexible, autonomous engagement does not. However, the negative unique association of harmonious passion requires longitudinal confirmation before it can guide practice.

## 5 Limitations and future directions

Several limitations should be acknowledged. First, gambling-related luck beliefs in this study were assessed using the luck subscale of the Gambling Attitudes and Beliefs Survey (GABS-23). Although this instrument has been widely used and validated across different countries and gambling populations (73–75), its factor structure has been questioned recently. Studies have recovered multidimensional solutions in which luck items tend to merge with gambler’s fallacy items rather than forming a distinct factor (34, 76). Our luck scores may therefore reflect a broader set of chance-related distortions rather than pure luck beliefs. Future studies should replicate our findings using alternative instruments, such as the Gambling-Related Cognitions Scale (77) and the gambling-specific Personal Luck Usage Scale (53). A related issue concerns the measurement of general luck beliefs. Limitations of Darke and Freedman’s unidimensional scale have prompted reconceptualization (78, 79), culminating in a bidimensional model that distinguishes between belief in luck as a deterministic force and belief in being personally lucky—two discrete and uncorrelated components (80). This model accounts for lottery gambling variance beyond personality (33). How these two general luck beliefs relate to gambling-specific luck beliefs, and subsequently to problem lottery gambling, remains to be explored.

Second, we did not consider the heterogeneity of lottery gambler types. Our sample included players of number games, scratch cards, and sports betting, but we did not analyze these groups separately. The associations among luck beliefs, skill beliefs, and problem gambling vary by game type, yet the empirical evidence has been mixed. Strategic gamblers tend to attribute outcomes to skill rather than luck (81, 82). However, one study found that strategic problem gamblers reported stronger personal luck perceptions than their non-strategic counterparts (51). A more recent study further observed that perceived personal luck was unrelated to problem gambling among strategic gamblers but was associated with it among mixed gamblers (83). Future research should examine gambling-related luck beliefs and skill beliefs jointly across gambler types, and test whether the mediation model identified in this study holds within each type.

Third, the cross-sectional design prevents causal inference. All variables were measured once by self-report, so the mediations we report are statistical rather than temporal. Reversed or reciprocal paths are plausible. Longitudinal designs are needed to trace the chain from financially focused self-concept to problem lottery gambling across time. Ecological momentary assessment offers a complementary tool. It can capture luck beliefs, passion states, and gambling episodes in daily life as they unfold, which reduces recall bias and reveals within-person processes (84). Relatedly, the negative unique association between harmonious passion and problem lottery gambling was observed only after accounting for obsessive passion. Whether this pattern reflects a protective process or statistical suppression cannot be determined from cross-sectional data and warrants longitudinal or experimental investigation.

Fourth, all constructs were assessed using self-report instruments in a single session. Although we applied procedural remedies and conducted Harman’s single-factor test, these remedies and diagnostics cannot conclusively rule out common method variance (85). Self-report data are also susceptible to recall errors and social desirability bias (86). In gambling contexts, participants may underreport their gambling involvement or minimize symptoms of problem gambling, and retrospective reports of gambling behavior are prone to memory distortion. Such biases may affect observed associations among study variables. To reduce reliance on a single data source, future studies would benefit from multi-source evidence, such as lottery purchase records, family-member reports, or behavioral tracking data (87).

Finally, given that the data were collected anonymously, we were unable to identify participants with elevated problem gambling scores or to provide them with responsible gambling information. Future studies should consider incorporating such safeguards.

## 6 Conclusion

This study examined whether gambling-related luck beliefs and lottery gambling passion sequentially mediate the association between financially focused self-concept and problem lottery gambling among Chinese lottery gamblers. Correlation analyses showed that financially focused self-concept was positively correlated with gambling-related luck beliefs, obsessive passion, harmonious passion, and problem lottery gambling. The structural equation model further revealed that financially focused self-concept was not directly associated with problem lottery gambling after accounting for the mediating variables, but showed significant indirect associations through gambling-related luck beliefs, obsessive passion, and harmonious passion. Serial indirect associations through luck beliefs and obsessive passion and through luck beliefs and harmonious passion were also significant. Obsessive passion exhibited the strongest positive indirect association, whereas harmonious passion exhibited a negative indirect association. Given the measurement limitations of the luck subscale, the unexamined heterogeneity of gambler types, and the cross-sectional design, future studies should replicate the findings with refined instruments, examine the model across gambler types, and adopt longitudinal designs to establish temporal ordering.

## Data Availability

The raw data supporting the conclusions of this article will be made available by the authors, without undue reservation.
