# Supplementary material for: Financially focused self-concept and problem lottery gambling: the chain mediating role of gambling-related luck beliefs and lottery gambling passion
Source: Front Psychiatry. 2026 Oct 14;17:1978508. doi: 10.3389/fpsyt.2026.1978508 (PMC13713455; doi:10.3389/fpsyt.2026.1978508)
Supplement: Supplementary file 1 [file Table1.docx]

TABLE S1. Standardized factor loadings of the measurement model.

| **Construct** | **Item** | **Standardized loading** |
| --- | --- | --- |
| Financially focused self-concept | FFS1 | 0.792 |
|  | FFS2 | 0.859 |
|  | FFS3 | 0.867 |
|  | FFS4 | 0.898 |
| Gambling-related luck beliefs | LUCK1 | 0.686 |
|  | LUCK2 | 0.838 |
|  | LUCK3 | 0.807 |
|  | LUCK4 | 0.724 |
| Harmonious passion | HP1 | 0.788 |
|  | HP2 | 0.809 |
|  | HP3 | 0.885 |
|  | HP4 | 0.790 |
|  | HP5 | 0.861 |
| Obsessive passion | OP1 | 0.905 |
|  | OP2 | 0.947 |
|  | OP3 | 0.954 |
|  | OP4 | 0.920 |
|  | OP5 | 0.901 |
| Problem lottery gambling | PG1 | 0.706 |
|  | PG2 | 0.743 |
|  | PG3 | 0.763 |
|  | PG4 | 0.886 |
|  | PG5 | 0.845 |
|  | PG6 | 0.888 |
|  | PG7 | 0.925 |
|  | PG8 | 0.895 |
|  | PG9 | 0.909 |

*Note.* All loadings were statistically significant at *p* < 0.001. For each latent factor, one item served as the marker indicator, with its unstandardized loading fixed to 1 for model identification.
